# Quantitative Analysis of Propofol Dosage in Cannabis Users: A Systematic Review and Meta-Analysis

**DOI:** 10.3390/jcm14030858

**Published:** 2025-01-28

**Authors:** Maxwell B. Baker, Dhanesh D. Binda, Ala Nozari, Joseph M. Kennedy, Erin Dienes, William E. Baker

**Affiliations:** 1 Department of Anesthesiology, Boston University Chobanian & Avedisian School of Medicine, Boston, MA 02118, USA; maxb98@bu.edu (M.B.B.); ddb96@bu.edu (D.D.B.);; 2 Larner College of Medicine, University of Vermont, Burlington, VT 054052, USA; 3 Department of Anesthesiology, Montefiore Einstein Medical Center, Bronx, NY 10467, USA; 4 Department of Emergency Medicine, Larner College of Medicine, Burlington, VT 05405, USA; joseph.kennedy@uvmhealth.org (J.M.K.); william.e.baker@uvm.edu (W.E.B.)

**Keywords:** cannabis use, propofol, anesthetic management, pharmacological interactions, perioperative care, anesthetic dosage, tetrahydrocannabinol

## Abstract

**Background**: Rising cannabis use poses significant challenges in the administration of general anesthetics, particularly propofol, due to potential alterations in pharmacodynamics caused by tetrahydrocannabinol and its interactions with central nervous system receptors. This systematic review and meta-analysis aims to consolidate the existing literature to quantify propofol requirements in cannabis users, highlighting the complex relationship between cannabis use and anesthetic management. **Methods**: A systematic search of English-language literature was conducted to identify studies with data on propofol dosing in adult cannabis users. Propofol requirements were defined as the total intraoperative dose needed to achieve and maintain adequate sedation or anesthesia, assessed using parameters like monitoring and procedural tolerance. A random-effects model was used with DerSimonian–Laird estimations for pooled effect sizes and 95% confidence intervals. Heterogeneity was assessed using I^2^ and Cochran’s Q statistics, and sensitivity analysis was conducted by grouping publications by design, size, and quality. **Results**: Eight qualified studies were identified with 2268 patients included. Patients who used cannabis were typically younger and more likely to smoke tobacco than non-users. Propofol requirements were significantly higher in cannabis users, who required an average additional dose of 47.33 mg compared to non-users. Subgroup analyses revealed that cannabis users undergoing general anesthesia needed an additional 30.57 mg intraoperatively, while those undergoing sedation for endoscopic procedures required an additional 53.02 mg. **Conclusions**: These results underscore the need for personalized anesthetic plans to accommodate physiological variations in cannabis users. However, the lack of standardized definitions for propofol requirements and the heterogeneity across studies necessitate caution in interpretation. The observed increase in propofol requirements suggests altered central nervous system sensitivities and receptor changes in cannabis users, emphasizing the need for further research to establish clear definitions, elucidate underlying mechanisms, and refine clinical guidelines for anesthetic management in this population.

## 1. Introduction

The increasing use of cannabis has the potential to significantly impact anesthesia practices, particularly due to its psychoactive cannabinoid (CB) component tetrahydrocannabinol (THC). In the United States (U.S.), over 15% of the population consumes cannabis [1,2]. In 2021, 19% of Americans aged 12 and older reported cannabis use, rising to a record 44% among adults aged 19 to 30 in 2022 [3,4]. This trend is reflected among preoperative patients as well, with recent studies reporting high rates of cannabis use in this patient population [5]. The pharmacodynamics between prominent cannabinoids in cannabis and many anesthetic agents remain underexplored. For instance, the amount of propofol required for sedation in cannabis users is an area of growing research. Propofol (2,6-diisopropylphenol), first synthesized in 1973 and introduced as an anesthetic in 1977, is a sedative-hypnotic agent widely used for inducing and maintaining general anesthesia (GA) [6,7]. Its mechanism of action primarily involves positive modulation of the inhibitory function of the neurotransmitter gamma-aminobutyric acid (GABA) through GABA-A receptors, hyperpolarizing neurons and enhancing inhibitory neurotransmission in the central nervous system to produce sedation and hypnosis.

Cannabinoids act primarily through G protein-coupled receptors, namely CB1 in the central and peripheral nervous systems and CB2 in lymphoid and hematopoietic cells [8]. Tetrahydrocannabinol functions as a partial agonist at both receptors. Cannabinoids also interact with other targets like transient receptor potential cation channel subfamily V member 1 (TRPV1), opioid, N-methyl-D-aspartate (NMDA), and GABA receptors. Regular cannabis users may experience changes in drug metabolism due to effects on liver enzymes such as cytochrome P450 (CYP). These interactions can alter phase I oxidative metabolism in the liver, affecting the metabolism of propofol and potentially increasing the required dosage during procedures to maintain desired sedation.

Propofol’s interaction with cannabis may complicate anesthetic management. Various studies have demonstrated an increased propofol requirement for sedation in rodents that were administered THC [9], and published case reports describe cannabis users requiring higher propofol doses for adequate sedation [10,11,12,13,14]. Several studies have investigated propofol dosing and cannabis use in humans; however, clinical observations vary due to differences in study designs and confounding factors. For example, Twardowski et al. found regular cannabis users required higher doses of fentanyl, midazolam, and propofol during endoscopic procedures [15], while Gangwani et al. reported no significant impact of THC on anesthetic requirements for various drugs during intravenous procedures [16]. Similarly, Yeung et al. and Flisberg et al. found no significant difference in the minimum alveolar concentration (MAC) of volatile gas or propofol doses for bispectral index (BIS) values, though Flisberg et al. observed a higher propofol dose was needed for laryngeal mask airway (LMA) insertion [17,18]. Zhang et al. observed generally higher propofol induction doses in cannabis users, suggesting a potential need for increased propofol dosing [19].

Physicians administering anesthetic agents such as anesthesiologists and emergency medicine physicians should consider a patient’s cannabis use history in their pre-procedure interview and postoperative care [20,21,22]. Despite the growing body of research, there remains a significant gap in quantifying the amount of propofol required for cannabis users during procedures. This review aims to synthesize the existing literature, drawing on insights from clinical studies that explore perioperative considerations for cannabis users.

## 2. Materials and Methods

### 2.1. Search Strategy

Two medical librarians performed a comprehensive search of multiple databases after consultation with the lead authors and a Medical Subject Heading (MeSH) analysis (The Yale MeSH Analyzer, http://mesh.med.yale.edu (accessed on 6 December 2024)) of key articles provided by the research team. In each database, an iterative process was used to translate and refine the searches. Only English-language articles were included due to authors’ lack of ability to read non-English academic writing or access to funds for translation. No date limitations were applied to the search. The formal search strategies used relevant controlled vocabulary terms and synonymous free-text words and phrases to capture the concepts of adult cannabis use and propofol requirements. The databases searched were MEDLINE (Ovid Epub Ahead of Print, In-Process, and In-Data-Review and Other Non-Indexed Citations; Daily and Versions 1946 to October 2023); Embase (Ovid 1974–2023); Web of Science Core Collection (Clarivate, 1982–2023); ProQuest Theses and Dissertations; OIAster; Google Scholar; Cochrane Library; and ClinicalTrials.gov. All searches were performed on 20 November 2023. The Ovid MEDLINE search strategy is available in the Supplementary Materials. The final searches retrieved a total of 3013 references, which were pooled in EndNote™ (version 21, Clarivate Analytics, Philadelphia, PA, USA) and de-duplicated to 2537 references. This set was uploaded to the Covidence© review software (Covidence, Melbourne, Australia, 2024), where an additional 97 duplicates were found.

### 2.2. Inclusion and Exclusion Criteria

**Figure 1:**
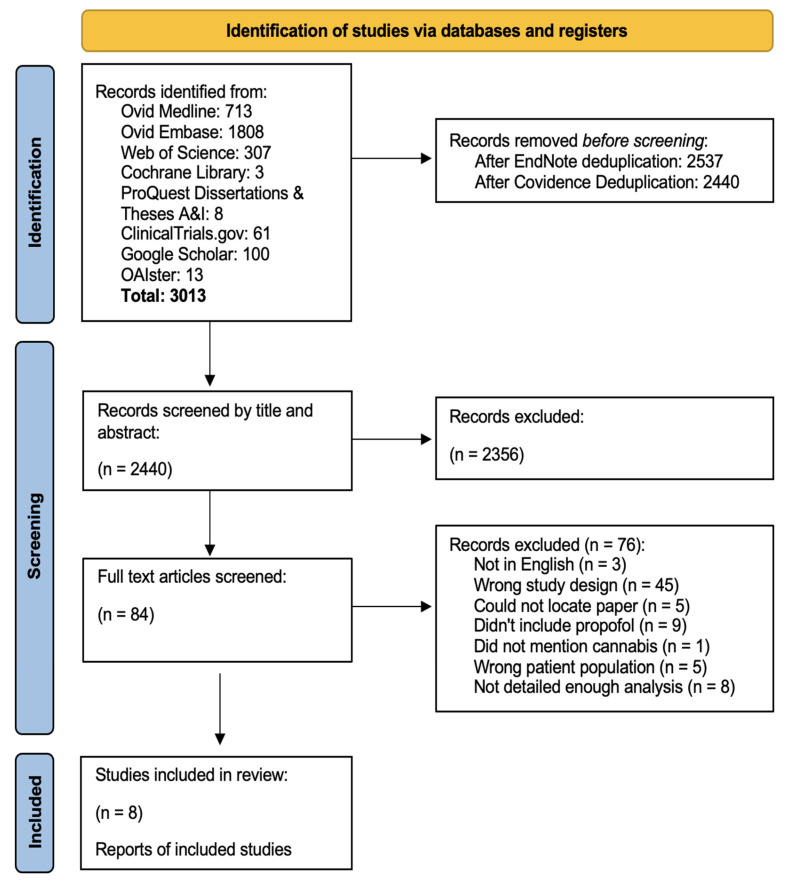
PRISMA flowchart for a systematic review of the effects of cannabis use on propofol requirements, conducted on 20 November 2023.

Peer-reviewed published original materials in English were included. Excluded were studies on pediatric populations, non-human subjects, those with fewer than 10 participants, and non-English publications. Our primary focus concentrated on studies reporting propofol requirements in adult patients who use cannabis, defined by indirect information recorded in the patient’s electronic medical record, patient-facing surveys, or THC metabolite urine screens. Within Covidence, two independent reviewers screened a total of 2440 records by title and abstract, and 2356 records were excluded. After screening each record, the two reviewers independently assessed the remaining 84 full-text articles for eligibility. At the time of the full-text review, one record representing a poster session (Kosirog et al.) became a peer-reviewed manuscript [23]. The same inclusion/exclusion criteria as above were applied, and a total of 76 articles were excluded. Any disagreements were resolved through consultation with a third reviewer. A total of eight articles were included in the study for data extraction. The systematic review protocol is reported according to the Preferred Reporting Items for Systematic Reviews and Meta-Analyses (PRISMA) Guidelines and was registered with PROSPERO (CRD42024484145, 31 January 2024) (Figure 1) [24].

### 2.3. Data Extraction

The data extracted included the author, publication year, study design, sample size, mean age, sex distribution, and key findings. For our analysis, we defined propofol requirements as the total dose of propofol administered intraoperatively to achieve and maintain adequate sedation or anesthesia. This was determined using clinical and physiological parameters such as processed EEG monitoring (e.g., BIS) and procedural tolerance, including successful airway placement or the absence of patient movement in response to noxious stimuli. Additional variables related to cannabis use, such as the frequency and method of consumption, and anesthesia outcomes, including propofol dosage, induction, and recovery times, were also collected. Where available, data on postoperative outcomes involving adverse events and recovery times were recorded.

### 2.4. Data Analysis

Similar studies were grouped for analysis. We used a random-effects model with DerSimonian–Laird estimations for pooled effect sizes and 95% confidence intervals (CIs). Forest plots summarize the results. Heterogeneity was assessed using I^2^ and Cochran’s Q statistics, with a *p*-value of < 0.05 indicating significant heterogeneity. A sensitivity analysis was conducted by grouping publications by design, size, and quality. The analysis was performed using the statistical software R, version 4.4.0 [25].

### 2.5. Risk of Bias Assessment

Publication bias was evaluated using funnel plots, the Begg rank correlation test, and the Egger regression test. We applied the Newcastle–Ottawa Scale for observational studies. Studies were categorized as good, fair, or poor quality based on AHRQ standards, considering criteria such as selection, comparability, and outcome assessment (Supplementary Materials).

## 3. Results

### 3.1. Study Characteristics

There were eight studies included in this systematic review (Figure 1), ranging from 2009 to 2024, of which the majority were published in 2020 or later [18,23,26,27,28,29,30,31]. There were six retrospective studies [26,27,28,29,30,31] and two prospective studies (Table 1) [18,23].

Of the eight studies included in the meta-analysis, eight (100%) asked patients to self-report their cannabis use [18,23,26,27,28,29,30,31], with one (12.5%) also employing a urine THC metabolite screen [26]. Out of the eight studies, two (25%) excluded patients with ASA scores > 2 [18,31] and three (37.5%) reported all ASA categorizations (Table 2) [28,29,30]. In the eight studies, nine independent populations contained the necessary information to conduct a meta-analysis, encompassing a total of 2268 subjects [18,23,26,27,28,29,30,31].

Among the studies included in the meta-analysis, cannabis users tended to be younger (*p =* 0.0017) and were more likely to smoke tobacco products than cannabis non-users (*p* < 0.0001) (Supplementary Materials). Cannabis use did not affect the average procedure time (*p =* 0.8178). However, the mean procedure times in Holmen et al. were over six times longer than in any other study (Table 2) [27]. The percentage of male participants varied widely between studies but only differed between cannabis users and non-users in three of the studies (*p =* 0.1510) [26,28,29]. 

### 3.2. Statistical Analysis

**Figure 2:**
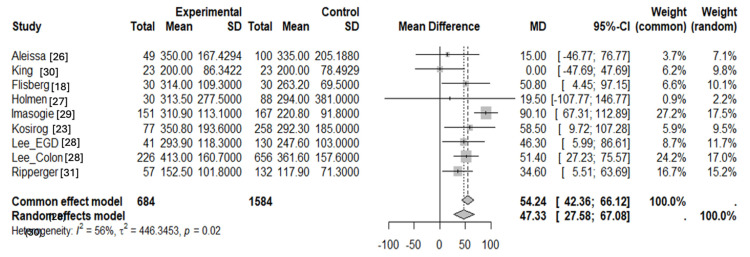
Meta-analysis showing the effect of cannabis use on propofol requirements during procedures [18,23,26,27,28,29,30,31].

The meta-analysis synthesized data from eight studies containing nine independent study populations to assess the effect of cannabis use on the average amount of propofol (mg) required during procedures (Table 2). Propofol requirements varied across studies, reflecting differences in patient populations, procedural types, and definitions used for adequate sedation. For instance, some studies relied on subjective clinical endpoints (e.g., no patient movement), while others utilized objective measures like BIS monitoring. The pooled analysis revealed a mean difference of 47.33 mg (95% CI: 27.58 to 67.08), indicating an approximately 47 mg dose increase for cannabis users versus non-users (Figure 2). Heterogeneity was assessed with the I^2^ statistic and Cochran’s Q, which both showed a significant amount of heterogeneity (*I*^2^ = 56%; *Q* = 18.2; *p* = 0.0198).

**Figure 3:**
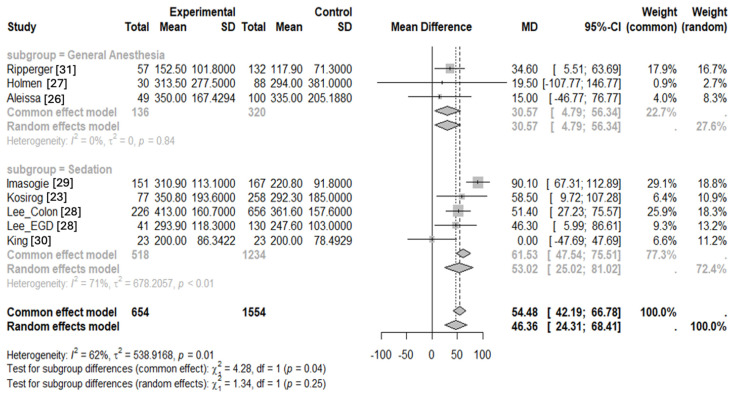
Sensitivity analysis showing the effect of cannabis use on propofol requirements intraoperatively during general anesthesia and sedation for endoscopic procedures separately (Flisberg et al.’s work is not included as this study investigated propofol required for laryngeal mask airway placement rather than sedation required for a procedure) [14,23,26,27,28,29,30,31].

Since the eight studies encompassed a variety of procedures, we conducted a subgroup analysis to determine if cannabis users undergoing GA [26,27,31] required different amounts of propofol intraoperatively compared to those receiving sedation for endoscopic procedures [23,28,29,30]. Both subgroups continue to show a positive mean difference in propofol dose between cannabis users and non-users (Figure 3). The estimated mean difference in propofol dose for patients undergoing GA was 30.57 mg (95% CI: 4.79 to 56.34), while those receiving procedural sedation was 53.02 mg (95% CI: 25.02 to 81.02). Under a random effects model, the differences in the subgroups were not statistically significant (*p* = 0.25).

To assess the robustness of our findings and the influence of various factors on the overall results of the meta-analysis, we conducted a sensitivity analysis. We examined the impact of heterogeneity and publication bias by conducting subgroup analyses based on sample sizes and study designs. To account for methodological differences, we grouped studies into prospective (*n* = 2) and retrospective (*n* = 6) designs (Supplementary Materials). The pooled mean difference in propofol requirements was higher in prospective studies (54.45 mg; 95% CI: 20.85 to 88.05) compared to retrospective studies (44.15 mg; 95% CI: 19.20 to 69.09). Under a random effects model, the differences in the subgroups were not statistically significant (*p* = 0.63). All subgroups continued to show a positive mean difference in propofol dose between cannabis users and non-users (Supplementary Materials). We assessed the certainty of evidence for our primary outcome using the GRADE framework. The certainty was rated as low due to inconsistencies across study methodologies, potential biases in retrospective data, and indirectness in measuring cannabis exposure (e.g., reliance on self-reported use). Furthermore, the high I^2^ value (56%) indicates moderate heterogeneity among the included studies.

### 3.3. Risk of Bias Findings

The funnel plot shows a slight asymmetry with an excess of studies with extreme effect sizes. The Begg rank correlation test (*p* = 0.4042) and the Egger linear regression test (*p* = 0.1245) suggest a lack of publication bias (Supplementary Materials). However, it is important to note that due to the limited number of studies included in this meta-analysis (*n* = 8), these tests may lack power. According to the Newcastle–Ottawa Scale, Flisberg et al., Kosirog et al., Lee et al., Imasogie et al., King et al., and Ripperger et al., received an overall quality rating of good. Aleissa et al. and Holmen et al. received a quality rating of fair (Supplementary Materials).

## 4. Discussion

In our systematic review, cannabis use criteria differed among the included studies; however, it was primarily determined through self-reporting (87.5%), with only one study using urine THC metabolite screening (Table 1). The meta-analysis of the eight included studies revealed that cannabis users tended to be younger, heavier, and more likely to smoke tobacco compared to non-users. However, cannabis use did not significantly affect the average procedure time. A notable finding was that cannabis users required approximately 47 mg of more propofol during procedures compared to non-users. When separated by type of procedure, cannabis users undergoing GA required 31 mg of more propofol intraoperatively while cannabis users receiving sedation for endoscopic procedures required 53 mg of more propofol. Given the low certainty of evidence, our findings should be interpreted with caution. The variability in study quality and cannabis exposure assessment underscores the need for more robust, prospective studies with standardized definitions of cannabis use and propofol requirements. While the data suggest a trend toward increased propofol requirements in cannabis users, these findings are hypothesis-generating and should guide future research rather than be considered definitive.

### 4.1. Physiological Mechanisms

Our meta-analysis found that cannabis use can significantly impact the dosing of propofol required for anesthesia. The physiological mechanisms underlying the interaction between cannabis use and propofol requirements are complex and not yet fully understood. Cannabis, containing psychoactive cannabinoids like THC, may alter the central nervous system’s sensitivity to anesthetics, including GABA-a agonists such as propofol. This alteration could manifest in varied requirements for propofol dosing [32]. Additionally, the chronic use of cannabis might lead to changes in receptor sensitivities, potentially affecting the pharmacodynamics of propofol with several receptor systems in addition to cannabinoid receptors, including TRPV1, opioid, NMDA, and GABA receptors. Anesthesia may also extend the metabolism time of cannabinoids due to their interference with CYP3A metabolism, altering phase I oxidative metabolism in the liver [33]. Another proposed mechanism suggests that propofol increases anandamide (endocannabinoid) levels in the brain by inhibiting the enzyme fatty acid amide hydrolase (FAAH), which typically terminates anandamide’s activity [34]. These mechanisms underscore the need for tailored anesthetic approaches for cannabis users [20]. Further research into these physiological interactions is crucial for refining anesthesia practices. The interaction between cannabis and propofol can lead to increased effects on breathing, heart rate, and blood pressure [35], necessitating close monitoring and dosage adjustments.

### 4.2. Clinical Implications

To assess the causal relationship between cannabis use and increased propofol requirements, we considered Bradford Hill’s criteria. While the strength of association is supported by a consistent increase in propofol requirements across studies, the criteria for temporality, biological gradient, and experimental evidence remain weak due to limited data. The consistency of findings across subgroups, however, aligns with the criterion of coherence. Future studies with better control of confounding factors and objective measurements of cannabis exposure will be essential to strengthen these causal inferences.

The findings of our review suggest that a history of cannabis use must be a critical consideration in determining appropriate propofol dosing, ensuring the effective and safe administration of anesthetics. The variability in cannabis consumption methods and frequency necessitates careful consideration when building anesthesia plans tailored to the individual [36]. To address the variability introduced by differing baseline propofol doses across studies, we chose to report absolute differences in milligrams rather than percentages. This approach prioritized clinical relevance, minimized variability caused by baseline differences, and ensured comparability across studies with diverse populations and procedures included in the meta-analysis.

Enhanced preoperative assessments should include detailed and structured inquiries into cannabis use, encompassing the frequency, duration, and potency of the products used, as well as the route of administration [37]. Such assessments are essential, as different methods of cannabis consumption (e.g., smoking cannabis flower, vaping potent extracts, edible ingestion, or sublingual tinctures) result in varying systemic and pharmacological effects [38]. For instance, patients who smoke cannabis flower occasionally are likely to have lower blood concentrations of THC than those who frequently consume highly potent THC extracts or concentrates. Additionally, complications arising from the route of cannabis use must also be considered. For example, chronic smoking is associated with increased airway reactivity, inflammation, and respiratory secretion production, all of which may complicate airway management during anesthesia [39,40].

Cannabis users are more prone to experiencing adverse perioperative events, including bronchospasm, laryngospasm, tachycardia, and elevated myocardial oxygen demand, particularly under conditions of high-stress anesthesia or insufficient premedication [32,36,41]. Inhaled cannabis, in particular, is linked to increased bronchial secretions and heightened airway reactivity. These risks necessitate proactive measures, such as the administration of glycopyrrolate or other anticholinergic agents, to effectively manage respiratory secretions effectively and reduce the likelihood of intraoperative complications. Furthermore, the use of inhaled short-acting beta-2 agonists or anticholinergic medications can be considered for cannabis users with a history of reactive airway disease or chronic bronchitis [41]. Preoperative optimization with these agents could mitigate airway-related risks and improve patient outcomes.

In addition to airway considerations, cannabis use has systemic effects that may alter hemodynamic stability during anesthesia. THC is known to affect the autonomic nervous system, often causing tachycardia and fluctuations in blood pressure. These changes can increase myocardial oxygen demand, potentially complicating management in patients with underlying cardiovascular conditions. Close intraoperative monitoring of vital signs and cardiovascular function is therefore critical, particularly in high-risk patients [42]. Anesthetic plans should also account for the potential need of higher doses of vasoactive agents to maintain hemodynamic stability in this population. Moreover, the potential impact of cannabis use extends beyond the intraoperative phase. Postoperative monitoring is equally important, as cannabis use may delay recovery times and increase the risk of postoperative nausea and vomiting (PONV), particularly in patients undergoing higher doses of propofol or other anesthetic agents. Multimodal approaches to PONV management, including the use of serotonin receptor antagonists (e.g., ondansetron) and dexamethasone, should be considered [43]. Cannabis users may also exhibit altered pain thresholds, which could influence their postoperative pain management requirements [44,45]. Preoperative consultation with a pain management specialist may be beneficial for developing tailored analgesic strategies that consider the patient’s cannabis use history.

The implications of these findings for clinical practice are far-reaching. Anesthesiologists and perioperative teams must integrate this knowledge into standard care protocols to optimize anesthetic care for cannabis users. This includes revising preoperative assessment protocols to incorporate targeted questions about cannabis use and its specific patterns. Additionally, the development of clinical guidelines that address the unique needs of cannabis users is essential. Recently, the American Society of Regional Anesthesia and Pain Medicine (ASRA Pain Medicine) released guidelines for managing perioperative patients who use cannabis, emphasizing the importance of universal preoperative screening to identify cannabis use and its impact on anesthesia [46]. The guidelines recommend delaying surgery in cases of acute intoxication, tailoring anesthesia plans based on cannabis use and addressing postoperative pain management challenges in chronic users, while underscoring the need for nonjudgmental communication to ensure accurate patient disclosures and enhance safety. Moreover, training programs for physicians, residents, and perioperative staff should also include modules on the perioperative implications of cannabis use. These programs can enhance awareness of the physiological effects of cannabis, potential complications, and evidence-based management strategies. Incorporating simulated scenarios involving cannabis-using patients may further prepare anesthesiology teams to respond effectively to the unique challenges posed by this population.

### 4.3. Public Health, Multidisciplinary Collaboration, and Economic Implications

The increasing prevalence of cannabis use, particularly in regions where it has been legalized for recreational or medicinal purposes, underscores its growing public health relevance in the perioperative setting [47]. This trend translates to an ever-larger subset of patients presenting for surgical procedures with cannabis-related physiological changes that can influence anesthetic management. The variability in cannabis potency, routes of administration, and user behaviors introduces unique challenges that necessitate a collaborative, multidisciplinary approach to ensure patient safety and optimize outcomes [37]. For instance, anesthesiologists must work closely with primary care physicians, emergency medicine physicians and pain management specialists to obtain accurate substance-use histories and tailor preoperative and postoperative care plans accordingly. Surgeons also play a key role, particularly in counseling patients on how cannabis use may affect recovery, pain control, and the risk of complications. Pharmacologists can also contribute by elucidating drug interactions and advising on adjusted anesthetic dosages or alternative regimens.

From a public health perspective, it is crucial to address the potential disparities in access to care that cannabis users may face. Stigma surrounding cannabis use, even in legalized regions, could lead to underreporting during preoperative assessments, hindering anesthesiologists’ ability to provide optimal care. Public health campaigns could aim to destigmatize discussions about cannabis use in medical settings, emphasizing the importance of full disclosure for patient safety [48]. Furthermore, the high prevalence of cannabis use among patients with chronic pain, mental health conditions, or socioeconomic challenges highlights the need for culturally aware and accessible healthcare approaches, especially in rural communities [49,50,51,52]. These public health efforts must be supported by robust clinical research to inform evidence-based guidelines and refine anesthetic practices for this growing patient population.

The cost and resource implications of cannabis use in the perioperative setting are significant. Increased anesthetic requirements for cannabis users, as demonstrated by the need for higher propofol dosages, can lead to escalated drug costs and environmental impacts over time [53]. While individual cases may incur only modest increases in drug expenditures, the cumulative financial impact on healthcare systems is likely to be substantial as cannabis use becomes more widespread. Additionally, cannabis-related complications, such as prolonged recovery times or the increased risk of PONV, may require extended stays in recovery units, thereby straining hospital resources and increasing operational costs [54,55]. Patients with cannabis-induced airway reactivity or cardiovascular instability may necessitate additional monitoring or interventions, further contributing to resource utilization [56].

To address these challenges, healthcare systems should invest in staff training and infrastructure improvements tailored to the needs of cannabis users. Enhanced preoperative assessments, specialized equipment for managing airway complications, and protocols for close postoperative monitoring are vital. Furthermore, interdisciplinary collaboration among anesthesiologists, pain specialists, public health experts, and policymakers will be critical in creating cost-effective strategies to manage this unique patient population. Leveraging technology, such as decision-support tools within electronic medical records, could also help standardize care and reduce variability in anesthetic management for cannabis users [57]. Ultimately, a multidisciplinary, systems-level approach is necessary to ensure that healthcare systems can adapt effectively to the growing prevalence of cannabis use while maintaining high standards of patient care and cost efficiency.

### 4.4. Limitations of This Review

This systematic review has several limitations. The potential for selection bias exists due to specific inclusion criteria and the reliance on certain databases, possibly limiting the generalizability of the findings due to the small sample size. While meta-analyses often include 10 or more studies [58], the paucity of research on this topic allowed for the inclusion of eight. The variability in study designs, methodologies, and quality among the included studies may have impacted the consistency and reliability of the results. The observed differences in propofol requirements between prospective and retrospective studies highlight potential methodological biases, including variability in data collection, patient recall of cannabis use, and study design limitations. One significant potential point of variability affecting data synthesis was the inconsistency in reporting and classifying cannabis use (frequency, dosage, and method) across studies. Several studies classified subjects as cannabis users or non-users based on indirect information recorded in the patient’s electronic medical record, while others used both patient-facing surveys and THC metabolite urine screens for accurate group placement. Similarly, the definition of “propofol requirement” may have varied among studies, as this subjective measure was based on the need for adequate sedation for specific procedures and/or airway management. Furthermore, not all studies explicitly reported the use of BIS monitoring to assess sedation or anesthesia depth. Some relied on alternative measures, such as clinical endpoints, to determine adequate sedation. This lack of consistent BIS monitoring is a notable limitation that may have affected the comparability of findings across studies.

The absence of long-term follow-up data in many studies might not fully represent the prolonged effects of cannabis on propofol requirements. Another limitation could involve how the response to propofol may depend on whether patients are acutely intoxicated during procedures compared to those who are chronic users. Acute intoxication may result in altered pharmacokinetics and pharmacodynamics of propofol, potentially leading to unpredictable sedation outcomes, increased risks of adverse effects, or the need for dosage adjustments. On the other hand, chronic users may develop tolerance, necessitating higher doses during procedures. Additionally, cigarette smoking, which independently increases propofol requirements, presents another confounding factor [59]. Cannabis users in the included studies were more likely to smoke cigarettes, yet data on the independent effects of smoking on propofol dosing were inconsistently reported. While some studies adjusted for tobacco use, this was not uniform across all analyses. Finally, by focusing solely on English-language publications, important research in other languages may have been overlooked, leading to publication bias. Finally, evolving cannabis genetics contributes to varying cannabinoid potencies, and geographical variations in cannabis types could affect the applicability of our review’s findings in different contexts.

### 4.5. Directions for Future Research

Future research in the field of cannabis use and anesthetic administration should emphasize well-designed, controlled longitudinal studies to better understand the long-term effects of cannabis on anesthesia outcomes, beyond the immediate impacts observed when administering sedative medications. While much of the current evidence is derived from observational studies and small-scale trials, robust prospective research is critical to establish causative relationships and account for confounding variables, such as cigarette smoking. Given the established impact of smoking on propofol requirements, future studies should include detailed assessments of tobacco use and its independent effects to distinguish these from those of cannabis. By collecting data on smoking status, frequency, and duration, researchers can better control for this confounder and clarify the interplay between cannabis and tobacco in determining anesthetic dosing.

Such studies should follow patients over time to evaluate not only intraoperative factors but also postoperative recovery, long-term complications, and overall patient outcomes. Subgroup analyses should be prioritized, focusing on specific patient demographics, including age, sex, comorbidities, and cannabis-use patterns. For example, older adults who use cannabis medicinally for chronic conditions may have different physiological responses compared to younger recreational users. Similarly, the type of surgical procedure may influence how cannabis interacts with anesthetic agents. High-stress procedures, such as cardiac or thoracic surgeries, may exacerbate cannabis-induced cardiovascular effects, whereas shorter, less invasive procedures might show fewer complications. By tailoring research to these subpopulations, clinicians can develop more precise and individualized anesthetic protocols.

The exact mechanisms by which cannabinoids, particularly THC and CBD, interact with anesthetic agents like propofol remain poorly understood. Research exploring the molecular biology of these interactions could yield critical insights into their effects on drug efficacy, receptor binding, and metabolism. For instance, differing cannabinoids’ interactions with CB1 and CB2 receptors, as well as their influence on GABAergic, NMDA, and opioid pathways, may alter the pharmacodynamics of propofol. Additionally, studies should examine how chronic cannabis use affects the upregulation of liver enzymes, particularly those involved in hepatic phase I oxidative metabolism such as cytochrome P450 isoforms, and whether these changes lead to altered propofol metabolism and clearance. Such investigations could provide the basis for targeted pharmacological interventions and improve patient safety.

The evolution of cannabis genetics and processing methods introduces another area of significant research potential. Modern cannabis products often contain extremely high levels of cannabinoids, especially ∆9-THC, and are available in diverse forms, including edibles, drinks, sublingual oils, water-soluble tinctures, vaporizer cartridges, concentrates, and even topical lotions. Each method of consumption has unique pharmacokinetics that may influence anesthetic requirements differently. Studies should investigate how these variations impact propofol dosing and efficacy, as well as whether certain cannabis products pose a higher risk of perioperative complications. For example, inhaled cannabis may exacerbate airway reactivity, while ingested forms of potent extract may have delayed or prolonged effects on drug interactions.

In addition, future research should explore how cannabis use affects the efficacy of other anesthetic agents, including volatile gases, regional anesthetics, and multimodal pain management regimens. For instance, a recent retrospective cohort study found that older adults with documented cannabis use received statistically higher inhalational anesthetic concentrations during general anesthesia compared to non-users, but the clinical significance of this difference remains uncertain [60]. Comparing propofol to other intravenous anesthetics, such as etomidate or ketamine, in cannabis users could yield valuable insights into whether these alternatives offer advantages in specific clinical scenarios. Finally, the integration of cannabis biomarkers into clinical practice could enhance research and perioperative care. Studies should investigate the feasibility and accuracy of using blood, urine, or saliva tests to quantify recent cannabis exposure and correlate these levels with anesthetic requirements and outcomes. Furthermore, the incorporation of standardized monitoring tools like BIS could enhance comparability and precision in evaluating propofol requirements. These approaches would allow for more objective assessments and would facilitate the development of predictive models for anesthetic dosing in cannabis users.

## 5. Conclusions

This systematic review and meta-analysis highlight the complex relationship between cannabis use and propofol requirements, emphasizing the need for tailored sedation/anesthesia approaches. Our findings indicate that cannabis users may require higher doses of propofol during procedures. However, the low certainty of evidence and heterogeneity across studies necessitate caution in interpreting these results. The interaction between cannabis, especially its psychoactive component THC, and propofol suggests that cannabis users may have altered sensitivities and receptor changes in the central nervous system, affecting the pharmacodynamics of propofol. Rather than drawing definitive conclusions, these findings highlight the need for further research to explore the underlying mechanisms and clinical implications of cannabis use on anesthetic management.

## Figures and Tables

**Table 1:** Characteristics of included studies. THC, tetrahydrocannabinol; N/A, not available.

Study	Design	Sample Size	Procedure	Procedure Length (Min)	Cannabis Use Criteria	Frequency of Cannabis Use	Other Significant Outcomes
Flisberg, 2009 [18]	Prospective observational single center	60	Day-case general anesthesia with laryngeal mask	N/A	Regular use at least once per week for at least the past 6 months	1 day/week × 6 months	N/A
Aleissa, 2020 [26]	Retrospective single center	149	Total knee or total hip Arthroplasty	N/A	Social history of marijuana use within 6 months or + THC screen on admission	N/A	Higher postop opioid usage and higher postop pain scores in the cannabis group, and higher NSAID use in the controls.
Holmen, 2020 [27]	Retrospective single center	118	Isolated tibia open reduction and internal fixation	N/A	Self-reported in the month prior to surgery	N/A	The average total volume of sevoflurane administered was significantly higher among the cannabis-user group.
Lee, 2020 [28]	Retrospective single center	882	Colonoscopy	21.1–22.9	Daily use for more than 3 months	Daily × 3 months	N/A
			Esophagogastroduodenoscopy	7–9			
King, 2021 [30]	Retrospective single center	46	Esophagogastroduodenoscopy	N/A	Three primary documents for verbal self-report of cannabis use: (1) the preprocedural history and physical examination findings, (2) the nursing intake form, and (3) the pre-anesthesia assessment	N/A	N/A
Imasogie, 2021 [29]	Retrospective single center	318	Colonoscopy and/or esophagogastroduodenoscopy	N/A	Any duration of self-reported inhaled cannabis exposure	Daily (4/7 days × 1 week), weekly (1–2 days/week × weeks), monthly (1–2 times/month × 9 months), or occasional (<1 × 2 months)	N/A
Ripperger, 2023 [31]	Retrospective single center	189	Extraction of at least 2 teeth requiring general anesthesia	15–40	Any self-reported regular use of cannabis to provider	N/A	Cannabis users received significantly more midazolam, ketamine, and fentanyl than non-users.
Kosirog, 2024 [23]	Prospective observational single center	976	Colonoscopy and/or esophagogastroduodenoscopy	N/A	Patient survey prior to the endoscopy which addressed marijuana usage and frequency	N/A	N/A

**Table 2:** Characteristics of cannabis and non-cannabis users in the included studies. LMA, laryngeal mask airway; BMI, body mass index; COPD, chronic obstructive pulmonary disease; OSA, obstructive sleep apnea; SD, standard deviation; N/A, not available.

		Cannabis Users												Non-Cannabis Patients									
Study	Propofol (mg) Required (Mean ± SD)	Mean Age (Mean ± SD)	Procedure Length	Sample Size (n)	Female Sex (%)	Mean Weight (Mean ± SD)	BMI	Largest ASA Groups	Prior Smoking History (%)	Alcohol Use (%)	Narcotic Use (%)	Respiratory Disease (%)	Propofol (mg) Required (Mean ± SD)	Mean Age (Mean ± SD)	Procedure Length	Sample Size	Female Sex (%)	Mean Weight (Mean ± SD)	BMI	Largest ASA Groups	Smoking History (%)	Narcotic Use (%)	Respiratory Disease (%)
Flisberg, 2009 [18]	Total induction dose for LMA insertion 314.0 ± 109.3	28.0 ± 8.0	N/A	30	0	80.7 ± 12.4	N/A	ASA I and ASA II	N/A	N/A	Excluded	N/A	Total induction dose for LMA insertion 263.2 ± 69.5	22.0 ± 9.0	N/A	30	0	78.9 ± 12.5	N/A	ASA I and ASA II	N/A	Excluded	N/A
Aleissa, 2020 [26]	Intraoperative propofol used 350.0 ± 167.4	56.7 ± 12.4	N/A	49	38.7	N/A	29.5 ± 6.3	N/A	N/A	N/A	Excluded	N/A	Intraoperative propofol used 335.0 ± 205.2	66.8 ± 10.2	N/A	100	62	N/A	31.1 ± 5.9	N/A	N/A	Excluded	N/A
Holmen, 2020 [27]	Intraoperative propofol used 313.5 ± 277.5	N/A	166	30	N/A	N/A	N/A	N/A	N/A	No significant difference between groups	Excluded	N/A	Intraoperative propofol used 294.0 ± 381.0	N/A	165	88	N/A	N/A	N/A	N/A	N/A	Excluded	N/A
Lee, 2020 Colon [28]	Total propofol used 413.0 ± 160.7	53.5 ± 13.9	22.9 ± 10.8	226	39.0	84.8 ± 21.4	28.0 ± 6.0	ASA II	14.6	N/A	N/A	N/A	Total propofol used 361.6 ± 157.6	60.1 ± 12.5	8.5 ± 5.5	656	51.4	79.9 ± 19.6	27.2 ± 5.7	ASA I and ASA II	3.5	Excluded	N/A
Lee, 2020 EGD [28]	Total propofol used 293.9 ± 118.3	47.3 ± 16.5	7.9 ± 3.8	41	61.1	80.7 ± 18.5	27.3 ± 5.2	ASA II	14.6	N/A	N/A	N/A	Total propofol used 247.6 ± 103.0	67.7 ± 11.3	22.8 ± 10.0	130	58.5	79.3 ± 18.2	29.1 ± 15.2	ASA II	5.4	Excluded	N/A
King, 2021 [30]	Total propofol used 200.0 ± 86.3	41.1 ± 14.2	5.7 ± 2.2	23	78.3	81.3 ± 17.6	28.4 ± 6.0	ASA II and ASA III	N/A	N/A	N/A	N/A	Total propofol used 200.0 ± 78.5	41.5 ± 14.7	5.7 ± 2.2	23	78.3	79.8 ± 17.5	29.7 ± 5.5	ASA II and ASA III	N/A	N/A	N/A
Imasogie, 2021 [29]	Total propofol used 310.9 ± 113.1	43.7 (18–71)	16.7 ± 10.9	151	30.4	82.9 ± 23.4	N/A	ASA II	68.9	N/A	17.9	COPD, asthma, or OSA: 25.8	Total propofol used 220.8 ± 91.8	53.8 (23–88)	18.3 ± 9.6	167	60.4	77.4 ± 17.2	N/A	ASA II	41.3	9.6	COPD, asthma or OSA: 16.8
Ripperger, 2023 [31]	Intraoperative propofol used 152.5 ± 101.8	26.6 ± 6.4	N/A	57	71.9	N/A	N/A	ASA I and ASA II	N/A	N/A	N/A	N/A	Intraoperative propofol used 117.9 ± 71.3	28.2 ± 7.8	N/A	132	72.7	N/A	N/A	ASA I and ASA II	N/A	N/A	N/A
Kosirog, 2024 [23]	Total propofol used 350.8 ± 193.6	57.7 ± 13.7	25.2 ± 17.1	210	71.9	N/A	28.2 ± 5.9	N/A	Smoking tobacco: 35.7; vape: 13.5	N/A	5.7	COPD: 10.9; OSA: 26.2	Total propofol used 292.3 ± 185.0	61.4 ± 12.7	24.1 ± 18.3	766	72.7	N/A	31.2 ± 6.4	N/A	Smoking tobacco: 19.0; vape: 3.7	4.9	COPD: 13.5; OSA: 40.2

## Data Availability

Data sharing is not applicable to this manuscript as no new data were created nor analyzed in this study.

## Supplementary Materials

The following supporting information can be downloaded at: https://www.mdpi.com/article/10.3390/jcm14030858/s1, Search syntax, PRISMA Checklist for Meta-Analyses, The Newcastle–Ottawa Quality Assessment Scale, Differences in demographic characteristics among participants, Sensitivity analysis by study design, Sensitivity analysis by sample size, and Publication bias funnel plot.

## Glossary of Terms

**THC**	Tetrahydrocannabinol
**CB**	Cannabinoid
**U.S.**	United States
**GA**	General anesthesia
**TRPV1**	Transient receptor potential cation channel subfamily V member 1
**NMDA**	N-Methyl-D-Aspartate
**GABA**	Gamma amino butyric acid
**CYP**	Cytochrome P450
**MAC**	Minimum alveolar concentration
**BIS**	Bispectral index
**LMA**	Laryngeal mask airway
**MeSH**	Medical subject heading
**PRISMA**	Preferred Reporting Items for Systematic Reviews and Meta-Analyses
**FAAH**	Fatty acid amide hydrolase
**ASRA Pain Medicine**	American Society of Regional Anesthesia and Pain Medicine
