# Supplementary material for: Quantitative Analysis of Propofol Dosage in Cannabis Users: A Systematic Review and Meta-Analysis
Source: J Clin Med. 2025 Jan 28;14(3):858. doi: 10.3390/jcm14030858 (PMC11818839; doi:10.3390/jcm14030858)

Appendix: Supplementary Data

Table of Contents

Section	Page Number
Search Syntax	11
PRISMA Checklist for Meta-Analyses	13-15
The Newcastle-Ottawa Quality Assessment Scale	16
Differences in demographic characteristics among participants	17
Sensitivity analysis by study design	18
Sensitivity analysis by sample size	19
Publication Bias Funnel Plot	20

Ovid MEDLINE Search Strategy

#	**Query**
1	exp Cannabis/
2	exp Marijuana Smoking/
3	exp Marijuana Abuse/
4	exp Medical Marijuana/
5	exp Cannabinoids/
6	exp Dronabinol/
7	(Cannabi* or Marijuana or THC or Dronabinol or Tetrahydrocannabinol).mp.
8	1 or 2 or 3 or 4 or 5 or 6 or 7
9	exp Propofol/
10	(Propofol or Diprivan or An#esthetic? or sedation?).mp.
11	exp Anesthetics, Intravenous/ or exp Conscious Sedation/ OR exp Deep Sedation/
12	9 or 10 or 11
13	8 and 12
14	limit 13 to english language

PRISMA 2020 Checklist[20]

Section and Topic	Item #	Checklist item	Location where item is reported
**TITLE**			
Title	1	Identify the report as a systematic review.	1
**ABSTRACT**			
Abstract	2	See the PRISMA 2020 for Abstracts checklist.	3
**INTRODUCTION**			
Rationale	3	Describe the rationale for the review in the context of existing knowledge.	2
Objectives	4	Provide an explicit statement of the objective(s) or question(s) the review addresses.	2
**METHODS**			
Eligibility criteria	5	Specify the inclusion and exclusion criteria for the review and how studies were grouped for the syntheses.	3
Information sources	6	Specify all databases, registers, websites, organisations, reference lists and other sources searched or consulted to identify studies. Specify the date when each source was last searched or consulted.	3
Search strategy	7	Present the full search strategies for all databases, registers and websites, including any filters and limits used.	2-3, Appendix
Selection process	8	Specify the methods used to decide whether a study met the inclusion criteria of the review, including how many reviewers screened each record and each report retrieved, whether they worked independently, and if applicable, details of automation tools used in the process.	2-3
Data collection process	9	Specify the methods used to collect data from reports, including how many reviewers collected data from each report, whether they worked independently, any processes for obtaining or confirming data from study investigators, and if applicable, details of automation tools used in the process.	2-3
Data items	10a	List and define all outcomes for which data were sought. Specify whether all results that were compatible with each outcome domain in each study were sought (e.g. for all measures, time points, analyses), and if not, the methods used to decide which results to collect.	2-4
	10b	List and define all other variables for which data were sought (e.g. participant and intervention characteristics, funding sources). Describe any assumptions made about any missing or unclear information.	2-4
Study risk of bias assessment	11	Specify the methods used to assess risk of bias in the included studies, including details of the tool(s) used, how many reviewers assessed each study and whether they worked independently, and if applicable, details of automation tools used in the process.	4
Effect measures	12	Specify for each outcome the effect measure(s) (e.g. risk ratio, mean difference) used in the synthesis or presentation of results.	2-4
Synthesis methods	13a	Describe the processes used to decide which studies were eligible for each synthesis (e.g. tabulating the study intervention characteristics and comparing against the planned groups for each synthesis (item #5)).	2-4
	13b	Describe any methods required to prepare the data for presentation or synthesis, such as handling of missing summary statistics, or data conversions.	2-4
	13c	Describe any methods used to tabulate or visually display results of individual studies and syntheses.	2-4

Section and Topic	Item #	Checklist item	Location where item is reported
	13d	Describe any methods used to synthesize results and provide a rationale for the choice(s). If meta-analysis was performed, describe the model(s), method(s) to identify the presence and extent of statistical heterogeneity, and software package(s) used.	2-4
	13e	Describe any methods used to explore possible causes of heterogeneity among study results (e.g. subgroup analysis, meta-regression).	2-4
	13f	Describe any sensitivity analyses conducted to assess robustness of the synthesized results.	2-4
Reporting bias assessment	14	Describe any methods used to assess risk of bias due to missing results in a synthesis (arising from reporting biases).	2-4
Certainty assessment	15	Describe any methods used to assess certainty (or confidence) in the body of evidence for an outcome.	2-4
**RESULTS**			
Study selection	16a	Describe the results of the search and selection process, from the number of records identified in the search to the number of studies included in the review, ideally using a flow diagram.	4-5
	16b	Cite studies that might appear to meet the inclusion criteria, but which were excluded, and explain why they were excluded.	4-5
Study characteristics	17	Cite each included study and present its characteristics.	4-5
Risk of bias in studies	18	Present assessments of risk of bias for each included study.	7
Results of individual studies	19	For all outcomes, present, for each study: (a) summary statistics for each group (where appropriate) and (b) an effect estimate and its precision (e.g. confidence/credible interval), ideally using structured tables or plots.	6-7
Results of syntheses	20a	For each synthesis, briefly summarise the characteristics and risk of bias among contributing studies.	6-7
	20b	Present results of all statistical syntheses conducted. If meta-analysis was done, present for each the summary estimate and its precision (e.g. confidence/credible interval) and measures of statistical heterogeneity. If comparing groups, describe the direction of the effect.	6-7
	20c	Present results of all investigations of possible causes of heterogeneity among study results.	6-7
	20d	Present results of all sensitivity analyses conducted to assess the robustness of the synthesized results.	6-7
Reporting biases	21	Present assessments of risk of bias due to missing results (arising from reporting biases) for each synthesis assessed.	6-7
Certainty of evidence	22	Present assessments of certainty (or confidence) in the body of evidence for each outcome assessed.	6-7
**DISCUSSION**			
Discussion	23a	Provide a general interpretation of the results in the context of other evidence.	7-9
	23b	Discuss any limitations of the evidence included in the review.	9
	23c	Discuss any limitations of the review processes used.	9
	23d	Discuss implications of the results for practice, policy, and future research.	9-10
**OTHER INFORMATION**			
Registration and protocol	24a	Provide registration information for the review, including register name and registration number, or state that the review was not registered.	3
	24b	Indicate where the review protocol can be accessed, or state that a protocol was not prepared.	3
	24c	Describe and explain any amendments to information provided at registration or in the protocol.	N/A
Support	25	Describe sources of financial or non-financial support for the review, and the role of the funders or sponsors in the review.	10

Section and Topic	Item #	Checklist item	Location where item is reported
Competing interests	26	Declare any competing interests of review authors.	10
Availability of data, code and other materials	27	Report which of the following are publicly available and where they can be found: template data collection forms; data extracted from included studies; data used for all analyses; analytic code; any other materials used in the review.	10

Note: N/A (*Not Assessed*).

For more information, visit: http://www.prisma-statement.org/

The Newcastle-Ottawa Quality Assessment Scale for risk of bias analysis for observational studies.

Study	Selection ★★★★	Comparability ★★	Outcome ★★★	Overall Score ★★★★★★★★★ (9)	Interpretation
Flisberg 2009[14]	★★★☆	★★	★★☆	★★★★★★★ (7)	Good quality
Aleissa 2020[22]	★★★☆	★★	☆★☆	★★★★★★ (6)	Fair quality
Holmen 2020[23]	★★★☆	☆☆	☆★☆	★★★★ (4)	Fair quality
Lee 2020[24]	★★★☆	★★	★★☆	★★★★★★★★ (8)	Good quality
Imasogie 2021[25]	★★★☆	★★	★★☆	★★★★★★★ (7)	Good quality
King 2021[26]	★★★☆	★★	★★★	★★★★★★★★ (8)	Good quality
Ripperger 2023[27]	★★★☆	★★	★★☆	★★★★★★★ (7)	Good quality
Kosirog 2024[19]	★★★☆	★★	★★☆	★★★★★★★ (7)	Good quality

Mean age, weight, and BMI as well as percent male participants and smokers in the included studies.

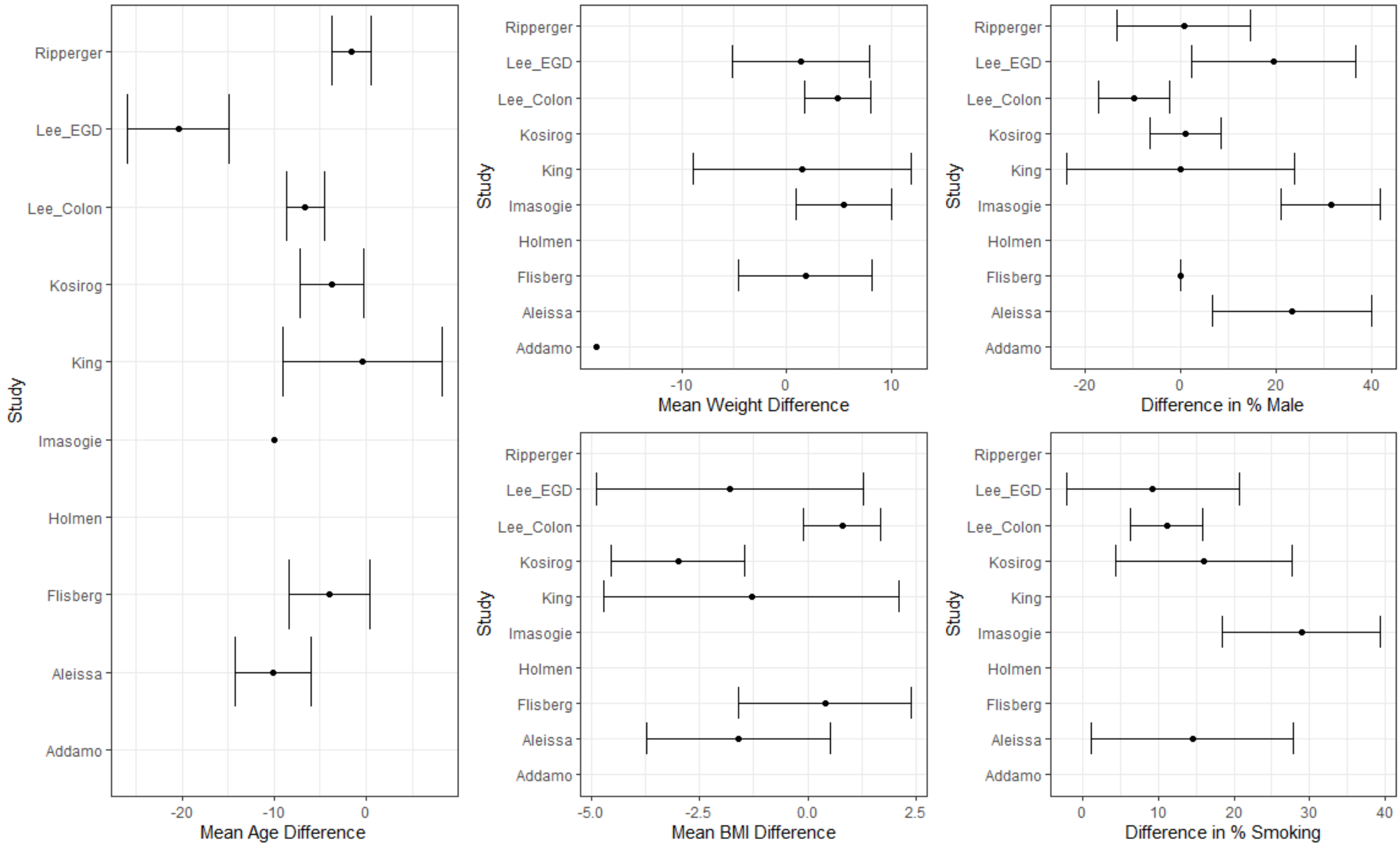

Sensitivity analysis by study design showing the effect of cannabis use on propofol requirements during procedures.

Study	Experimental Total	Experimental Mean	Experimental SD	Control Total	Control Mean	Control SD	MD	95%-CI	Weight (common)	Weight (random)
Retrospective										
Imasogie	151	310.90	113.1000	167	220.80	91.8000	90.10	[67.31; 112.89]	27.2%	17.5%
Lee_Colon	226	413.00	160.7000	656	361.60	157.6000	51.40	[27.23; 75.57]	24.2%	17.0%
Lee_EGD	41	293.90	118.3000	130	247.60	103.0000	46.30	[5.99; 86.61]	8.7%	11.7%
Ripperger	57	152.50	101.8000	132	117.90	71.3000	34.60	[5.51; 63.69]	16.7%	15.2%
Holmen	30	313.50	277.5000	88	294.00	381.0000	19.50	[-107.77; 146.77]	0.9%	2.2%
Aleissa	49	350.00	167.4294	100	335.00	205.1880	15.00	[-46.77; 76.77]	3.7%	7.1%
King	23	200.00	86.3422	23	200.00	78.4929	0.00	[-47.69; 47.69]	6.2%	9.8%
Common effect model	577			1296			54.21	[41.51; 66.91]	87.5%	.
Random effects model							44.15	[19.20; 69.09]	.	80.4%
Heterogeneity: $I^2 = 67\%$, $\tau^2 = 657.5451$, $p < 0.01$										
Prospective										
Kosirog	77	350.80	193.6000	258	292.30	185.0000	58.50	[9.72; 107.28]	5.9%	9.5%
Flisberg	30	314.00	109.3000	30	263.20	69.5000	50.80	[4.45; 97.15]	6.6%	10.1%
Common effect model	107			288			54.45	[20.85; 88.05]	12.5%	.
Random effects model							54.45	[20.85; 88.05]	.	19.6%
Heterogeneity: $I^2 = 0\%$, $\tau^2 = 0$, $p = 0.82$										
**Common effect model**	**684**			**1584**			**54.24**	**[42.36; 66.12]**	**100.0%**	**.**
**Random effects model**							**47.33**	**[27.58; 67.08]**	**.**	**100.0%**

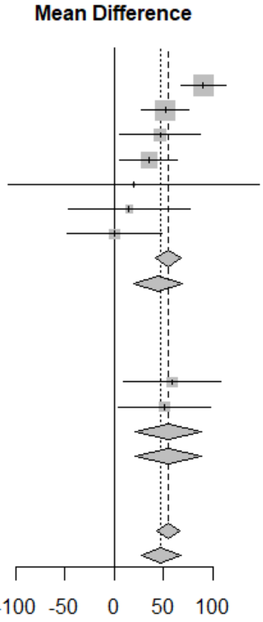

Heterogeneity: $I^2 = 56\%$, $\tau^2 = 446.3453$, $p = 0.02$
Test for subgroup differences (common effect): $\chi^2_1 = 0.00$, df = 1 ($p = 0.99$)
Test for subgroup differences (random effects): $\chi^2_1 = 0.23$, df = 1 ($p = 0.63$)

Sensitivity analysis by sample size showing the effect of cannabis use on propofol requirements during procedures.

Study	Experimental Total	Experimental Mean	Experimental SD	Control Total	Control Mean	Control SD	MD	95%-CI	Weight (common)	Weight (random)
**Small**										
Flisberg	30	314.00	109.3000	30	263.20	69.5000	50.80	[4.45; 97.15]	6.6%	10.1%
King	23	200.00	86.3422	23	200.00	78.4929	0.00	[-47.69; 47.69]	6.2%	9.8%
Common effect model	53			53			26.12	[-7.11; 59.36]	12.8%	.
Random effects model							25.72	[-24.06; 75.50]	.	19.9%
Heterogeneity: $I^2 = 55\%$, $\tau^2 = 714.7050$, $p = 0.13$										
**Moderate**										
Lee_EGD	41	293.90	118.3000	130	247.60	103.0000	46.30	[5.99; 86.61]	8.7%	11.7%
Ripperger	57	152.50	101.8000	132	117.90	71.3000	34.60	[5.51; 63.69]	16.7%	15.2%
Holmen	30	313.50	277.5000	88	294.00	381.0000	19.50	[-107.77; 146.77]	0.9%	2.2%
Aleissa	49	350.00	167.4294	100	335.00	205.1880	15.00	[-46.77; 76.77]	3.7%	7.1%
Common effect model	177			450			35.13	[13.42; 56.85]	29.9%	.
Random effects model							35.13	[13.42; 56.85]	.	36.2%
Heterogeneity: $I^2 = 0\%$, $\tau^2 = 0$, $p = 0.86$										
**Large**										
Imasogie	151	310.90	113.1000	167	220.80	91.8000	90.10	[67.31; 112.89]	27.2%	17.5%
Kosirog	77	350.80	193.6000	258	292.30	185.0000	58.50	[9.72; 107.28]	5.9%	9.5%
Lee_Colon	226	413.00	160.7000	656	361.60	157.6000	51.40	[27.23; 75.57]	24.2%	17.0%
Common effect model	454			1081			70.50	[54.81; 86.20]	57.3%	.
Random effects model							68.53	[41.09; 95.98]	.	44.0%
Heterogeneity: $I^2 = 63\%$, $\tau^2 = 348.3948$, $p = 0.06$										
**Common effect model**	**684**			**1584**			**54.24**	**[42.36; 66.12]**	**100.0%**	**.**
**Random effects model**							**47.33**	**[27.58; 67.08]**	**.**	**100.0%**

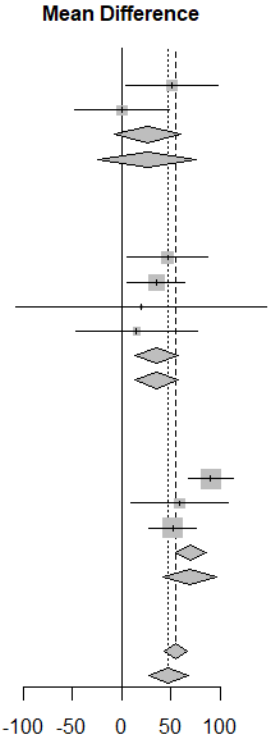

Heterogeneity: $I^2 = 56\%$, $\tau^2 = 446.3453$, $p = 0.02$
Test for subgroup differences (common effect): $\chi^2_2 = 9.85$, df = 2 ($p < 0.01$)
Test for subgroup differences (random effects): $\chi^2_2 = 4.19$, df = 2 ($p = 0.12$)

Funnel plot showing publication bias for the effect of cannabis use on propofol requirements during procedures.

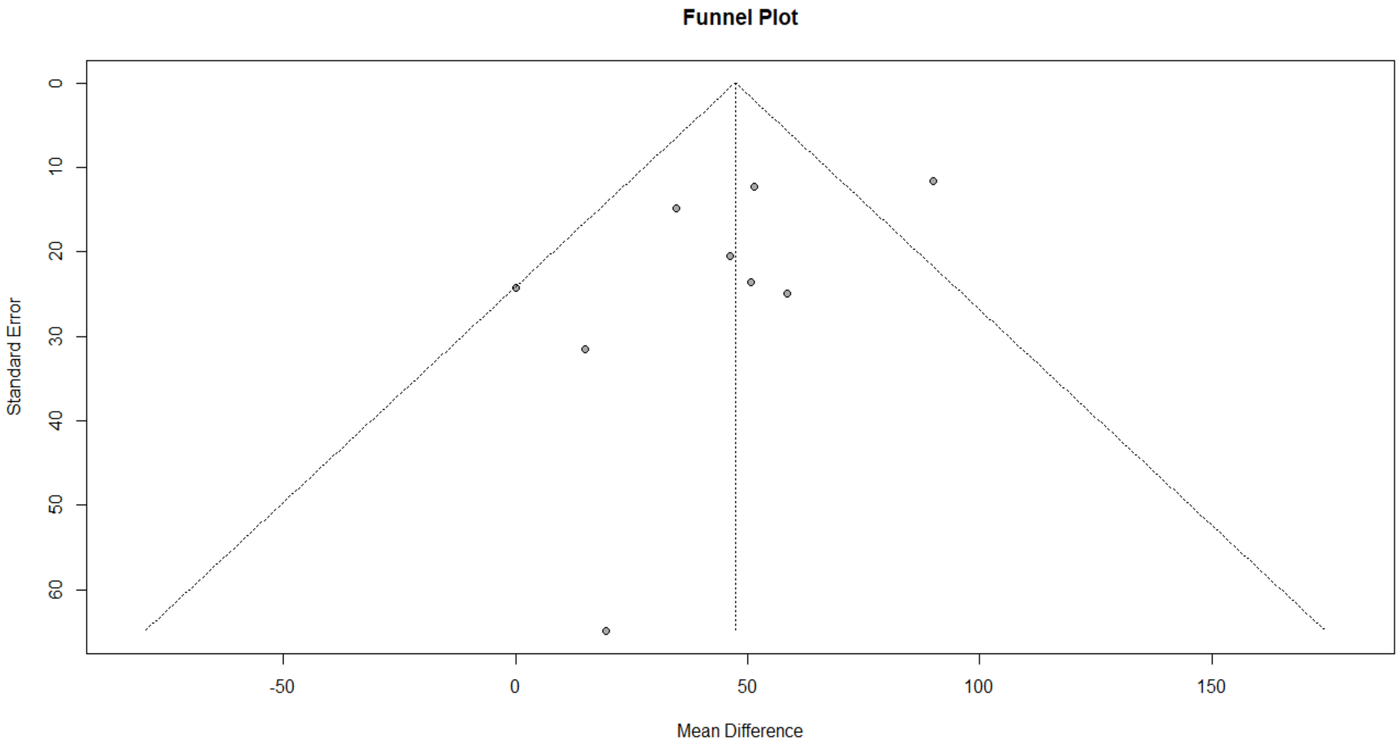

Supplement: Supplementary file 1 [file jcm-14-00858-s001.zip › jcm-3372679-supplementary.pdf]
